# Understanding intentions to use a multi-component supported self-management platform for long COVID-19: a mixed-methods evaluation in Hong Kong

**DOI:** 10.1038/s41598-025-01239-0

**Published:** 2025-05-08

**Authors:** Leonard Ho, Ming Hong Kwong, Ka Wai Yuen, Sam Ho Sum Yuen, Pui Chee Hung, Dexing Zhang, Samuel Yeung Shan Wong, Vincent Chi Ho Chung

**Affiliations:** 1 Jockey Club School of Public Health and Primary Care, Faculty of Medicine, The Chinese University of Hong Kong, Hong Kong Special Administrative Region, People’s Republic of China; 2 School of Public Health Building, Prince of Wales Hospital, Shatin, New Territories, Hong Kong Special Administrative Region, People’s Republic of China

**Keywords:** COVID-19, Self-management, Digital health, Behavioural intentions, Patient-centred care, Rehabilitation, Patient education

## Abstract

A culturally appropriate, multi-component self-management platform was developed to facilitate Long COVID-19 recovery in Hong Kong, comprising a smartphone application, a website, and a customer relationship management (CRM)-based messaging system. Using a mixed-methods design, we evaluated users’ intentions to utilise the platform through the behavioural attributes of the Meta-UTAUT (Meta-analysis-based modified Unified Theory of Acceptance and Use of Technology) framework. Structured interviews were conducted to explore themes influencing users’ intentions to use the platform, focusing on different attributes. Themes from the interviews were summarised using directed content and thematic analyses. These results informed the design of a cross-sectional survey quantifying the influence of those attributes on users’ utilisation of the platform. Multivariate logistic regressions were conducted to investigate the associations between sociodemographic and health characteristics and the likelihood of responses to each attribute. Analysis of 45 interviews identified 17 themes influencing platform use, across six attributes: performance expectancy, effort expectancy, social influence, facilitating conditions, perceived information security, and perceived enjoyment. The survey of 326 users revealed that each of these attributes influenced the intentions of over 80% of participants using the platform. Gender, age, educational attainment, and employment status were significantly associated with their responses to specific attributes. Supported self-management is an emerging intervention for Long COVID-19. While online platforms enhance access to health information, interactions through CRM-based messaging systems may optimise professional and emotional support, thereby improving user engagement. Resources may be directed towards alleviating digital barriers, particularly for older populations.

**Supplementary Information:**

The online version contains supplementary material available at 10.1038/s41598-025-01239-0.

## Introduction

Post-COVID-19 condition, more commonly known as “Long COVID-19”, is the continuation or development of new symptoms three months following the initial SARS-CoV-2 infection, and those symptoms last for at least two months without other explanations^1^ A meta-analysis revealed that over half of the individuals with Long COVID-19 would have a significantly poor quality of life attributable to persistent fatigue, dyspnoea, anosmia, sleep disturbances, and mental health issues^2^ The Office of National Statistics estimated that as many as 1.5 million people in the United Kingdom experiencing self-reported Long COVID-19 reported that the symptoms adversely affected their day-to-day activities^3^ About one-fifth of this population reported that they could not live alone without assistance and either had reduced ability to work or had to leave work altogether^3^.

Given the impacts of Long COVID-19 on personal lives and national productivity, various care models have been proposed and adopted by health and social care providers worldwide, with supported self-management being one of the most commonly applied principles^4^ Supported self-management empowers individuals with Long COVID-19 to pursue rehabilitation goals aimed at improving their quality of life, co-designed with self-management facilitators (e.g., health and social care professionals), while placing particular emphasis on the individuals’ lived experiences as evidence^5^ The National Institute for Health and Care Excellence (NICE) recommends supported self-management as an essential intervention for individuals with Long COVID-19 in the United Kingdom^6^ In its guidelines, NICE outlines five categories of advice and information that facilitators should provide to support self-management, including: (i) methods for self-managing Long COVID-19 symptoms; (ii) information on health and social care providers for further support with self-management; (iii) sources of additional support (e.g., support groups and social prescribing); (iv) guidance on accessing face-to-face health and social care services; and (v) resources on Long COVID-19 that individuals may share with their family, carers, and friends^6^ The World Health Organization’s Living Guidance for Clinical Management of COVID-19 also offers symptom-based self-management strategies for facilitators and individuals with Long COVID-19, enabling the settings of evidence-based rehabilitation goals^7^.

In response to the potentially high prevalence of Long COVID-19 (which was subsequently found to be 51.9% in 2023)^8^ and the growing need for self-management support in Hong Kong, we launched a free-of-charge, culturally appropriate smartphone app and website for the population in September 2022. The smartphone app is based on the COVID Recovery App developed by the Institute of Clinical Science and Technology (ICST) for the National Health Service Wales, aiming to support, motivate, and encourage physical and psychological progress towards recovery from Long COVID-19 symptoms and their impact through achievable milestones^9^ In collaboration with the ICST, we formally translated the textual and video content in the smartphone app from English to Cantonese, invited health and allied health professionals with relevant clinical expertise to review the translated content and produce culturally relevant and evidence-based elements (e.g., Chinese medicine), and arranged charity partners and laypersons to assess the comprehensibility of the translated content. A total of 130 videos have been produced, covering most of the common Long COVID-19 symptoms and providing symptom management advice. Of these, 90 videos are based on content created by National Health Service Wales, featuring local doctors and physiotherapists as speakers, while the remaining 40 videos are original, developed in collaboration with Chinese medicine practitioners from a local university. The app also includes signposting information for local face-to-face health and allied health services for Long COVID-19. Upon signing up, individuals can access information and videos on self-managing their symptoms, along with goal-setting tools to track progress and clinic details. The website has a user interface similar to the smartphone app and serves the same purpose. The website^10^ has a user interface similar to the smartphone app and serves the same purpose.

To enhance the accessibility of the supported self-management, we subsequently adopted a customer relationship management (CRM)-based approach to enable users to access our content via WhatsApp Messager (Meta Platforms, Menlo Park, California, United States). CRM systems have been used widely in customer services to maximise communication efficiency and quality, while minimising service costs, through exploiting automated scripting based on known solutions^11^ When an user initiates a conversation with us, our 24/7 chatbot will ask about their basic demographic information, as well as ongoing Long COVID-19 symptom(s) by providing a list of symptoms for them to choose from. The chatbot will then automatically send relevant self-management videos and textual information to the user, categorising the content based on demographic and clinical characteristics. We also broadcast information about new videos and health educational materials via WhatsApp Messenger, tailored to user categories. Voice messages and other inquiries that cannot be addressed by the chatbot are managed by our staff. This approach enables users to access our content and information through WhatsApp Messenger, offering several advantages: (i) no new application needs to be installed on users’ smartphones, given the widespread use of WhatsApp Messenger; (ii) account registration or new passwords are not required; (iii) chat records can be easily retained and accessed by authorised personnel; (iv) users can share content with others simultaneously; (v) stickers and emojis can be used to add a human touch to the conversation; and (vi) users with low IT skills can easily access our tips and information.

This study tests two hypotheses. First, we hypothesised that users’ intentions to utilise the multi-component supported self-management platform could be explained through the Meta-UTAUT (Meta-analysis-based modified Unified Theory of Acceptance and Use of Technology) framework,^12^ based on behavioural attributes (discussed below). To test this, our mixed-methods study assessed the intentions of Hong Kong users to use the Long COVID-19 supported self-management platform and its various components, focusing on specific behavioural attributes. Second, we hypothesised that sociodemographic and COVID-19-related characteristics would influence users’ intentions to use the platform. To explore this, the study examined the associations between these characteristics and the behavioural attributes using regression analyses.

## Methods

### Theoretical framework

Both the qualitative and quantitative phases of this study were guided by the Meta-UTAUT framework^12^ This modified framework, developed from the original UTAUT framework and 162 UTAUT-based empirical studies, aims to provide a comprehensive understanding of individuals’ intentions to adopt information technology^12^ The Meta-UTAUT framework includes 10 behavioural attributes: the original four core attributes (performance expectancy, effort expectancy, social influence, and facilitating conditions)^13^ and six additional behavioural and technological attributes (compatibility, perceived information security, perceived social pressure, personal innovativeness, resistance to change, and perceived enjoyment)^12^ These attributes have been shown to influence individuals’ acceptance and utilisation of information technology. Table S1, Supplemental material, presents the definitions for the Meta-UTAUT attributes. This mixed-methods study was based on an exploratory sequential design, where the structure of the quantitative phase was informed by the results in the qualitative phase^14^.

The study complied with the Declaration of Helsinki, and ethical approval was obtained from the Joint Chinese University of Hong Kong–New Territories East Cluster Clinical Research Ethics Committee (Reference number: 2022.268).

### Qualitative phase

#### Recruitment of interviewees

We recruited 45 current users of the Long COVID-19 supported self-management platform from the user database to participate in the semi-structured individual telephone interviews. The inclusion criteria were: (i) experience using any component (i.e., website, smartphone app, and CRM-based messaging system) of the platform; (ii) aged ≥ 18 years; (iii) proficiency in Cantonese; and (iv) capability to provide informed consent. Individuals with physical or mental conditions that limited their participation were excluded. The sample size of 45 fulfilled the number of interviewees expected to achieve saturation in qualitative research (*n* = 16–24)^15^. All interviewees were informed of the interview details and gave consent before data collection. An incentive of HKD300 cash coupons was offered after completing all required tasks.

#### Data collection

We prepared the interviews based on the Meta-UTAUT framework. We first asked the interviewees to describe their reasons for using the Long COVID-19 supported self-management platform and whether they thought they were the appropriate audience. These two questions were followed by specific prompts relating to the potential themes influencing their intentions to use the platform and its different components, covering all the Meta-UTAUT attributes. Subsequently, we invited them to comment on each component in terms of user-friendliness and person-centredness, as well as on the textual and video information regarding Long COVID-19 self-management in Chinese medicine and conventional medicine. Sociodemographic characteristics and COVID-19 infection history were also collected from all interviewees.

The interviews were conducted by authors with expertise in qualitative research (MHK, LH, VCHC). Each interview took approximately 45 min and was conducted between August 2023 and February 2024. Each interview was audio-recorded after receiving written informed consent from the interviewee.

#### Data analysis

We performed interviews, transcription, and data analysis concurrently to track emerging themes, which enabled timely follow-up questions for the next interview and clarifications with the interviewees. We transcribed the audio recordings verbatim and imported the transcripts into ATLAS.ti 7.11 (ATLAS.ti GmbH, Berlin, Germany) for data analysis in two steps as follows:

##### Step 1: coding of transcripts

The responses from the first 24 interviewees were coded independently by two authors (LH; MHK). The coding process involved: (i) reading the interviewees’ responses in the transcripts, (ii) considering their relevance to the definitions of the Meta-UTAUT attributes, and (iii) assigning these responses to one or more attributes via directed content analysis^16^ The two authors then compared their coding results and developed a consensus-based coding scheme to ensure the consistency of subsequent coding exercises. The authors coded the remaining transcripts and met frequently (after coding every five transcripts) to assess consistency and seek consensus. When the authors failed to reach a consensus on text interpretation, they discussed the matter with a senior author (VCHC).

##### Step 2: generation and rating of themes

After coding the data into the Meta-UTAUT attributes, one author (MHK) was responsible for generating themes related to the use of the Long COVID-19 supported self-management platform (and its three components) from the codes via thematic analysis^17^ Another author (LH) cross-checked and confirmed the themes and codes. When necessary, the two authors resolved any discrepancies through discussion. In a deliberated consensus process, the two authors then assigned a rating to each theme within each attribute based on the criteria reflecting its strength (degree of emphasis by the interviewees) and valence (positive or negative influence)^18^ The ratings ranged from − 2 to + 2, where − 2 refers to a strong negative influence on the use of the Long COVID-19 supported self-management platform and + 2 a strong positive influence. The themes that exhibited a strong positive or negative influence were classified as key themes and selected for further discussion.

### Quantitative phase

Considering the high penetration of smartphones in Hong Kong^19^ and the lack of discussion by the interview participants, we did not include the Meta-UTAUT attributes of compatibility, perceived social pressure, personal innovativeness, and resistance to change in the subsequent cross-sectional survey.

#### Recruitment of participants

From May 2024 to June 2024, we consecutively recruited 326 users of the Long COVID-19 supported self-management platform from the user database to participate in the cross-sectional survey. The inclusion criteria were: (i) experience using all components of the platform; (ii) aged ≥ 18 years; (iii) proficiency in Cantonese; and (iv) capability to provide informed consent. Individuals with physical or mental conditions that hindered their participation were excluded. All participants were informed of the survey details and provided consent before data collection. An incentive of HKD300 cash coupons was offered to participants who responded to all required survey items.

#### Data collection and analysis

This cross-sectional survey consisted of 42 items covering six of the 10 Meta-UTAUT attributes, aiming to measure the influence of different attributes on participants’ utilisation of the Long COVID-19 supported self-management platform and its components. The participants were required to rate on a five-point Likert scale, with 1 being strongly disagree and 5 being strongly agree. We illustrated item scores using medians and interquartile ranges (IQRs) and summed the scores of respective items for each attribute to generate attribute scores, which were also presented with medians and IQRs. The cut-off score for an attribute was calculated as the number of its items multiplied by the score of a neutral response (i.e., 3). For instance, the attribute of performance expectancy consisted of four items; therefore, its cut-off score was 12 (4 items × 3). Participants scoring more than 12 were deemed to have given a positive response to this attribute. In addition, participants’ sociodemographic characteristics and COVID-19 infection history were summarised using means and standard deviations or medians and IQRs, as appropriate.

We also performed independent multivariate logistic regressions to explore how sociodemographic characteristics and COVID-19 infection history predicted the likelihood of participants responding positively or negatively to each attribute. The results were presented as adjusted odds ratios (AORs) with 95% confidence intervals (CIs).

## Results

### Qualitative phase

#### Sociodemographic characteristics of the interviewees

The interviewees were predominantly female (68.9%), with the majority aged over 60 (42.2%). Over half of them had tertiary education or above (53.3%), while the remainder had secondary or primary-level education. A significant proportion of interviewees were retired (33.3%), with others employed in clerical, service, or technical roles (24.4%) or in management/professional positions (15.6%). Smaller groups included homemakers (8.9%), associate professionals (8.9%), self-employed individuals (2.2%), and unemployed individuals (6.7%). Table 1 presents the sociodemographic characteristics of the 45 interviewees in detail.

**Table 1:** Sociodemographic characteristics of the interview participants.

Sociodemographic characteristics	All participants (*N* = 45)
Gender	
Female	31 (68.9%)
Male	14 (31.1%)
Age, years	
< 31	3 (6.7%)
31–45	11 (24.4%)
46–60	12 (26.7%)
> 60	19 (42.2%)
Educational attainment	
Primary or below	3(6.6%)
Secondary	18 (40.0%)
Tertiary or above	24 (53.3%)
Occupation	
Associate professional	4 (8.89%)
Management/professional	7 (15.6%)
Retired	15 (33.3%)
Homemaker	4 (8.9%)
Self-employed	5 (2.2%)
Unemployed	3(6.7%)
Clerk/service/technician	11(24.4%)

#### Themes influencing the use of the long COVID-19 supported self-management platform and its components

**Fig. 1:**
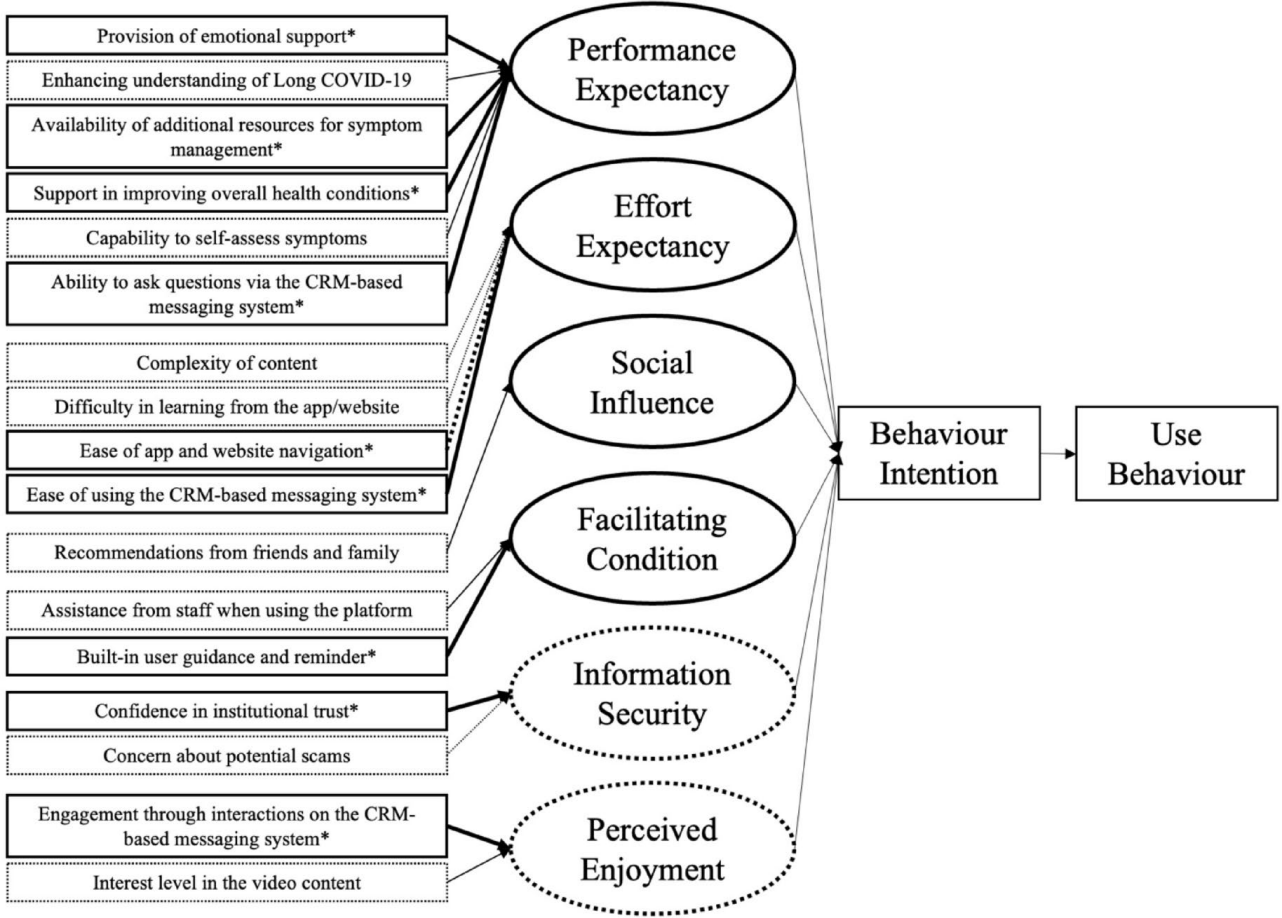
Themes derived from the interviews, summarised across the six Meta-UTAUT attributes, associated with the use of the Long COVID-19 supported self-management platform. Key themes are indicated by asterisks (*). Non-core factors (themes) are denoted by a dotted rectangle. Strength is indicated by the thickness of the arrow, while valence is shown by the type of arrow: normal arrow for positive influence and dotted arrow for negative influence. Solid circles represent core attributes, while dotted circles being other behavioural and technological attributes. *CRM* Customer relationship management, *Meta-UTAUT* Meta-analysis-based modified Unified Theory of Acceptance and Use of Technology.

Our qualitative data analysis yielded a total of 17 themes influencing the use of the Long COVID-19 supported self-management platform and its components across the six Meta-UTAUT attributes. Among these, eight exhibited a strong positive influence (i.e., + 2 in the strength and valence assessment), while one demonstrated a strong negative influence. Figure 1; Table 2 illustrates all the themes summarised from the interviews. The following section will describe the nine key themes in detail, accompanied by relevant quotes extracted from the transcripts.

**Table 2:** Themes influencing the use of the online long COVID-19 supported self-management platform and its components classified by Meta-UTAUT attributes.

Meta-UTAUT attributes	Themes identified from interviews (themes)	Strength and valence ratings^*^
Performance expectancy	Provision of emotional support	+ 2
	Enhancing understanding of Long COVID-19	+ 1
	Availability of additional resources for symptom management	+ 2
	Support in improving overall health conditions	+ 2
	Capability to self-assess symptoms	+ 1
	Ability to ask questions via the CRM-based messaging system	+ 2
Effort expectancy	Complexity of content	–1
	Difficulty in learning from the app/website	–1
	Ease of app and website navigation	–2
	Ease of using the CRM-based messaging system	+ 2
Social influence	Recommendations from friends and family	+ 1
Facilitating condition	Assistance from staff when using the platform	+ 1
	Built-in user guidance and reminder	+ 2
Information security	Confidence in institutional trust	+ 2
	Concern about potential scams	–1
Perceived enjoyment	Engagement through interactions on the CRM-based messaging system	+ 2
	Interest level in the video content	+ 1

*CRM* Customer relationship management, *Meta-UTAUT* Meta-analysis-based modified Unified Theory of Acceptance and Use of Technology.

^*^Strength refers to the degree of emphasis by the participants on the theme, and valence refers to its positive or negative influence. The ratings ranged from − 2 to + 2, where − 2 refers to a strong negative influence on the use of the online Long COVID-19 supported self-management platform and + 2 a strong positive influence.

##### Performance expectancy

*Provision of emotional support* Some interviewees reported emotional challenges during and after the pandemic, driven by anxiety over how to manage COVID-19 and Long COVID-19, as well as the emotional toll these conditions imposed. This distress was a key reason for seeking services from our online platform, beyond practical needs for managing Long COVID-19. Furthermore, the social impacts of the pandemic and its aftermath made it challenging for many interviewees to access help. The unprecedented and evolving nature of both COVID-19 and Long COVID-19 left them uncertain about the severity of their conditions and the most effective management strategies.

*“I thought I wouldn’t recover; I feared it would only get worse. But after hearing about this [programme]*,* I gained more confidence. There was less panic. I didn’t know anything before*,* so I panicked. It was a great relief that the doctors explained [the management of Long COVID-19] so clearly; it really alleviated a lot of my worries and depression. That*,* in turn*,* gave me the confidence to help myself. I felt that just by listening to the doctors*,* I was already halfway recovered. My condition isn’t as bad as I thought*,* so now I have the confidence to go for a full recovery… There is hope*,* there is hope!”* – Female user, over 60 years old.

*Availability of additional resources for symptom management* The concept of self-management for health conditions was relatively new in Hong Kong, and many interviewees were not fully aware of how to implement it. A significant proportion realised the objectives of the platform and wanted to seek additional resources or services for symptom management. Interestingly, due to the uncertainty about which services were appropriate for their specific Long COVID-19 conditions, many initially thought they could be referred to suitable healthcare providers via the platform. Despite their disappointment, they chose to continue receiving health information from the platform.

*“Yes*,* I was so surprised by the WhatsApp service [CRM-based messaging system]. I remember it was very late at the time*,* and I still got a response. I work in this industry myself*,* so I know we often set up chatbots for situations like this. I didn’t expect someone to reply so late. But when I asked my questions*,* I felt that the response was personal. They guided me to find other resources*,* which was very helpful because even though I can search online*,* sometimes I don’t find the right ones.”* – Female user, under 31 years old.

*Support in improving overall health conditions* Most interviewees approached the platform with the goal of seeking help in managing their Long COVID-19 symptoms. While some had specific objectives in mind—either self-managing their symptoms with the platform or seeking referrals to healthcare services—others were open to any approach that may yield satisfactory results. Many acknowledged that the platform aims to help enhance their overall health and did not expect it to provide a definitive cure for Long COVID-19.

*“I expect it [Long COVID-19] will be cured to some extent*,* though there’s a possibility that I may not fully recover. I’m not quite sure because I think my condition was quite severe. I do have the expectation that there will be improvement if I keep following the recommendations provided [by the platform]. However*,* if it happens*,* it will take a long time to see the results.”* – Female user, 31–45 years old.

*Ability to ask questions via the CRM-based messaging system* The major strength of the CRM component of the platform lied in its ability to facilitate inquiries. Given the complexities of Long COVID-19, many interviewees found it challenging to assess their own health conditions. They preferred using the CRM-based messaging system which allowed them to ask health-related questions, clarify their circumstances, and receive tailored solutions that better met their needs.

*“I didn’t ask any questions on WhatsApp at first. Then one day*,* your colleagues sent me some information about Long COVID-19 and asked if I wasn’t sleeping well. I replied ‘yes’*,* and they sent me some relevant information. That’s how I started regularly communicating with them*,* asking them questions regarding my condition.”* – Female user, 31–45 years old.

##### Effort expectancy

*Ease of smartphone app and website navigation* One of the most criticised aspects of the smartphone app and website was the difficulty interviewees experienced in locating relevant information. Some reported confusion about where to find details pertinent to their health conditions, and many struggled to navigate the smartphone app or website while trying to familiarise themselves with its features. Some interviewees found the content format challenging to consume. Overall, these navigation issues contributed to decreasing the use of the smartphone app and website.

*“Oh*,* the app overall… I don’t think it’s that simple. Every time I want to find out more [about a symptom or intervention]*,* I end up spending a lot of time trying to figure out what the information on the page is saying. I wish it were easier to understand and follow. The design feels a bit complicated*,* with so many videos embedded*,* and maybe the layout could be less tiresome.”* – Female user, over 60 years old.

*Ease of using the CRM-based messaging system*—In contrast to the difficulties interviewees face navigating the smartphone app and website, the CRM-based messaging system was frequently reported as intuitive and easy to use. Interviewees appreciated that the CRM system did not require registration, which could be troublesome for older adults or those with lower digital (or eHealth) literacy. Communicating about Long COVID-19 through the CRM system was akin to messaging their friends, reducing the need for interviewees to adapt to a new digital tool with which they had no prior experience.

*“It’s much easier to talk to my friends—if I want to say or ask something*,* I can just reach them on WhatsApp. In contrast*,* the information on the app is all there for me to see*,* but it might not necessarily be useful to me. If I could contact your colleagues directly via WhatsApp*,* I could ask for the information I need without having to register for the app and then spend a lot of time navigating it.”* – Male user, over 60 years old.

##### Facilitating condition

*Built-in user guidance and reminder* Many interviewees suggested incorporating user guidance in the smartphone app and website to help them navigate the complex layout and find relevant information. Furthermore, some highlighted the benefits of a reminder function within the app to assist in establishing a daily routine, particularly for recording daily Long COVID-19 symptoms. The inclusion of a chatbot was also proposed as a potential way to effectively guide users in navigating the app.

*“I have a busy schedule*,* so it would be better if the app had a reminder function. I would only follow the suggested interventions if I could remember to.”* – Female user, 31–45 years old.

*“The primary users of your platform are likely grassroots individuals or older adults. Even though they may have smartphones*,* they might only be familiar with basic functions. Therefore*,* I think it would be beneficial to add a chatbot to the app and website to help guide them in navigating the systems and finding relevant information.”* – Female user, under 31 years old.

##### Information security

*Confidence in institutional trust* One of the frequently mentioned themes in the interviews was the significance of trust in institutions developing online health management platforms, especially those with CRM-based messaging systems. Interviewees highlighted that the involvement of a local university and the largest charitable organisation in Hong Kong greatly enhanced their confidence in the platform. In addition, the reputation of the university’s medical school bolstered the credibility of the medical knowledge provided. Participants expressed scepticism toward information from private companies, citing concerns about potential conflicts of interest and the risk of personal information being compromised.

*“I knew the programme was hosted by (X) [local charitable organisation] and (Y) [a local university]*,* I would feel more comfortable. To be honest*,* in the online world*,* there are always risks when you share information. However*,* because it’s (X) and (Y)*,* I feel more at ease registering. If someone unknown asks me for information*,* I definitely won’t provide it.”* – Female user, over 60 years old.

##### Perceived enjoyment

*Engagement through interactions on the CRM-based messaging system* Another distinguishing element between the smartphone app or website and the CRM-based messaging system was the human interaction experienced by interviewees. Many expressed greater satisfaction when engaging with our staff, valuing the personal touch of these interactions. They reported feeling happy to receive responses to their messages and appreciated the sense of care shown towards their situations.

*“WhatsApp feels more humane. It really feels like someone on the other side is actually talking to me on WhatsApp and caring about my health.”* – Female user, over 60 years old.

### Quantitative phase

#### Sociodemographic characteristics and COVID-19 infection history of the participants

Table 3 displays the sociodemographic characteristics and COVID-19 infection history of the 326 participants. The majority were female (70.6%), with a mean age of 49.5 years and a mean body mass index of 22.4 kg/m^2^. Educational attainment varied, with 16.6% having junior secondary or less, 36.2% having senior secondary or vocational training, 12.6% having non-degree post-secondary education, and 34.7% having an undergraduate degree or higher. Over half (61.3%) were employed. Nearly half (48.5%) first contracted COVID-19 in 2022, and 66.9% had experienced only one infection by the time of the survey.

**Table 3:** Sociodemographic characteristics and COVID-19 infection history of the survey participants.

Sociodemographic characteristics and COVID-19 history	All participants (*N* = 326)
Gender	
Female	230 (70.6%)
Male	96 (29.4%)
Age, years	
Mean (SD)	49.5 (13.6)
Median (IQR)	49 (21)
Body mass index, kg/m^2^	
Mean (SD)	22.4 (3.6)
Median (IQR)	22.2 (5.1)
Educational attainment	
Junior secondary or below	54 (16.6%)
Senior secondary or vocational training	118 (36.2%)
Non-degree post-secondary	41 (12.6%)
Undergraduate or above	113 (34.7%)
Employment status	
Employed	200 (61.3%)
Unemployed	25 (7.7%)
Retired	64 (19.6%)
Homemaker	32 (9.8%)
Student	5 (1.5%)
Year of first COVID-19 infection	
2019	21 (6.4%)
2020	27 (8.3%)
2021	44 (13.5%)
2022	158 (48.5%)
2023	69 (21.2%)
2024	7 (2.1%)
Number of COVID-19 infection	
1	218 (66.9%)
2	85 (26.1%)
≥ 3	23 (7.1%)

*SD* Standard deviation, *IQR* Interquartile range.

#### Participants’ agreement on attribute scores

Table 4 presents the percentage of agreement among the participants for each item and attribute in the survey developed using the Meta-UTAUT framework. Performance expectancy showed a high positive agreement of 84.4%, indicating that participants were motivated by their expectation that the platform would aid their recovery and provide referrals to healthcare professionals. Effort expectancy received a similar level of agreement at 83.7%, suggesting that participants were encouraged by their expectation that the platform would be easy to use and its content comprehensible. Social influence had an agreement of 88.7%, showing that participants’ intentions to adopt the platform were influenced by peer or family recommendations and the developer’s reputation.

**Table 4:** Participants’ agreement on Meta-UTAUT items and attributes for the online long COVID-19 supported self-management platform and its components.

Meta-UTAUT attributes/items	Item level		Attribute level	
	Agree or strongly agree (*N* = 326)	Median (IQR)	Agree or strongly agree (*N* = 326)	Median (IQR)
Platform				
Performance expectancy			275 (84.4%)	15 (3)
Before joining, I expected this platform would be useful to my recovery	228 (69.9%)	4 (1)		
Before joining, I expected this platform would refer me to relevant healthcare professionals for consultations and treatments	182 (55.8%)	4 (1)		
This platform helps alleviate my symptoms	226 (69.3%)	4 (1)		
I will have a higher chance of recovering from Long COVID-19 if I continue to use this platform	225 (69.0%)	4 (1)		
Effort expectancy			273 (83.7%)	8 (1)
Before joining, I expected the content on this platform would be understandable	259 (79.4%)	4 (0)		
Before joining, I expected the tasks on this platform would be easy to follow	236 (72.4%)	4 (1)		
Social influence			289 (88.7%)	8 (1)
My family or friends think that I should use this platform	190 (58.3%)	4 (1)		
CUHK or HKJC has made me think that this platform is reliable	293 (89.9%)	4 (1)		
Website				
Perceived information insecurity			294 (90.2%)	12 (2)
I would only find Long COVID-19 or other health information on websites that I feel are safe	271 (83.1%)	4 (0)		
Generally speaking, the security level of those websites is enough for me to feel confident about finding Long COVID-19 or other health information on them	259 (79.4%)	4 (0)		
If I go to websites that are legitimate, I will receive accurate Long COVID-19 or other health information	269 (82.5%)	4 (0)		
Performance expectancy			277 (85.0%)	12 (2)
Before using, I expected this website would be useful to my recovery	261 (80.1%)	4 (0)		
Using this website helps alleviate my symptoms	226 (69.3%)	4 (1)		
I will have a higher chance of recovering from Long COVID-19 if I continue to use this website	234 (71.8%)	4 (1)		
Effort expectancy			278 (85.3%)	8 (1)
Before using, I expected this website would have clear and understandable instructions	255 (78.2%)	4 (0)		
Learning to use this website is easy for me	238 (73.0%)	4 (1)		
Facilitating conditions			265 (81.3%)	12 (2)
I have the knowledge necessary to use this website	232 (71.2%)	4 (1)		
This website is similar to other websites that I usually visit	219 (67.2%)	4 (1)		
Someone is available for assistance when I encounter difficulties when visiting this website	201 (61.7%)	4 (1)		
Perceived enjoyment			245 (75.2%)	8 (1)
It is fun to receive Long COVID-19 self-management information from this website	201 (61.7%)	4 (1)		
I enjoy receiving Long COVID-19 self-management information from this website	234 (71.8%)	4 (1)		
Smartphone app				
Perceived information insecurity			281 (86.2%)	12 (1)
I would only receive Long COVID-19 or other health information from smartphone apps that I feel are safe	260 (79.8%)	4 (0)		
Generally speaking, the security level of those smartphone apps is enough for me to feel confident about receiving Long COVID-19 or other health information from them	241 (73.9%)	4 (1)		
If I use to smartphone apps that are legitimate, I will receive accurate Long COVID-19 or other health information	255 (78.2%)	4 (0)		
Performance expectancy			266 (81.6%)	12 (2)
Before using, I expected this app would be useful to my recovery	246 (75.5%)	4 (0)		
Using this app helps alleviate my symptoms	222 (68.1%)	4 (1)		
I will have a higher chance of recovering from Long COVID-19 if I continue to use this app	214 (65.6%)	4 (1)		
Effort expectancy			269 (82.5%)	8 (1)
Before using, I expected this app would have clear and understandable instructions	244 (74.8%)	4 (0.75)		
Learning to use this app is easy for me	234 (71.8%)	4 (1)		
Facilitating conditions			262 (80.4%)	12 (2)
I have the knowledge necessary to use this app	235 (72.1%)	4 (1)		
This app is similar to other smartphone apps that I usually use	227 (69.6%)	4 (1)		
Someone is available for assistance when I encounter difficulties when using this app	197 (60.4%)	4 (1)		
Perceived enjoyment			240 (73.6%)	8 (2)
It is fun to receive Long COVID-19 self-management information from this app	189 (58.0%)	4 (1)		
I enjoy receiving Long COVID-19 self-management information from this app	230 (70.6%)	4 (1)		
CRM-based messaging system				
Perceived information insecurity			289 (88.7%)	12 (2)
I would only receive Long COVID-19 or other health information sent from organisations that I feel are reliable	268 (82.2%)	4 (1)		
Generally speaking, I feel confident about receiving Long COVID-19 or other health information from CRM-based messaging system	231 (70.9%)	4 (1)		
From legitimate organisations, I will receive accurate Long COVID-19 or other health information via CRM-based messaging system	255 (78.2%)	4 (0)		
Performance expectancy			271 (83.1%)	12 (2)
Before using, I expected this CRM-based messaging system would be useful to my recovery	250 (76.7%)	4 (0)		
Using this CRM-based messaging system helps alleviate my symptoms	223 (68.4%)	4 (1)		
I will have a higher chance of recovering from Long COVID-19 if I continue to use this CRM-based messaging system	229 (70.2%)	4 (1)		
Effort expectancy			272 (83.4%)	8 (2)
Before using, I expected this CRM-based messaging system would have clear and understandable instructions	252 (77.3%)	4 (0)		
Learning to use this CRM-based messaging system is easy for me	251 (77.0%)	4 (0)		
Facilitating conditions			274 (84.0%)	12 (2)
I have the knowledge necessary to use this CRM-based messaging system	259 (79.4%)	4 (0)		
Using this CRM-based messaging system is similar to chatting with friends and family via WhatsApp	253 (77.6%)	4 (0)		
Someone is available for assistance when I encounter difficulties when using this CRM-based messaging system	219 (67.2%)	4 (1)		
Perceived enjoyment			248 (76.1%)	8 (1)
It is fun to receive Long COVID-19 self-management information from this CRM-based messaging system	215 (66.0%)	4 (1)		
I enjoy receiving Long COVID-19 self-management information from this CRM-based messaging system	242 (74.2%)	4 (1)		

*CRM* Customer relationship management, *IQR* Interquartile range, *Meta-UTAUT* Meta-analysis-based modified Unified Theory of Acceptance and Use of Technology.

The participants mostly responded positively with perceived information security across different components, ranging from 86.2% for the smartphone app to 90.2% for the website. This implies that participants’ intentions to use the platform were driven by their confidence in the legitimacy of the service and the health information provided. Positive responses of at least 80% were also observed for performance expectancy, effort expectancy, and facilitating conditions across the components. Perceived enjoyment was slightly less influential on participants’ intentions to use the components, ranging from 73.6% for the smartphone app to 76.1% for the CRM-based messaging system, indicating that participants might not find receiving health information online enjoyable enough to increase their intentions to continue doing so.

##### Characteristics associated with meta-UTAUT attributes

The multivariable logistic regression analyses revealed that gender, age, educational attainment, employment status, the year of first COVID-19 infection, and the number of COVID-19 infections significantly predicted participants’ susceptibility to specific Meta-UTAUT attributes [Table 5]. Male participants tended to be motivated by effort expectancy to use the website (AOR 2.813; 95% CI 1.195–6.620). Older participants were unlikely to recognise the influence of facilitating conditions to use the website (AOR 0.965; 95% CI 0.937–0.994) and the CRM-based messaging system (AOR 0.972; 95% CI 0.946–0.998). They also inclined not to be driven by perceived enjoyment to use the website (AOR 0.972; 95% CI 0.946–0.998) and the CRM-based messaging system (AOR 0.970; 95% CI 0.945–0.996).

**Table 5:** Associations between sociodemographic characteristics and COVID-19 infection history and participants’ responses to Meta-UTAUT attribute: multivariate logistic regressions.

Attributes	Sociodemographic characteristics and COVID-19 infection history	Adjusted odds ratio (95% confidence interval)
Platform		
Performance expectancy	Recently infected by COVID-19	0.685 (0.495, 0.948)
	More COVID-19 infections	0.536 (0.342, 0.839)
Social influence	Higher educational attainment	1.847 (1.232, 2.770)
	Employed	11.167 (1.354, 92.110)
	More COVID-19 infections	0.486 (0.295, 0.802)
Website		
Effort expectancy	Male	2.813 (1.195, 6.620)
	Higher educational attainment	1.479 (1.064, 2.054)
Facilitating conditions	Older in age	0.965 (0.937, 0.994)
	More COVID-19 infections	0.637 (0.418, 0.971)
Perceived enjoyment	Older in age	0.972 (0.946, 0.998)
Smartphone app		
Perceived information insecurity	Higher educational attainment	1.625 (1.143, 2.310)
Performance expectancy	More COVID-19 infections	0.652 (0.427, 0.995)
Effort expectancy	Higher educational attainment	1.412 (1.045, 1.909)
Facilitating conditions	Higher educational attainment	1.438 (1.073, 1.927)
CRM-based messaging system		
Perceived information insecurity	Higher educational attainment	1.510 (1.045, 2.183)
Performance expectancy	More COVID-19 infections	0.644 (0.419, 0.990)
Effort expectancy	Higher educational attainment	1.375 (1.010, 1.872)
Facilitating conditions	Older in age	0.950 (0.922, 0.980)
	Higher educational attainment	1.530 (1.110, 2.110)
Perceived enjoyment	Older in age	0.970 (0.945, 0.996)

Only significant results are shown in the table.

*CRM* Customer relationship management, *Meta-UTAUT* Meta-analysis-based modified Unified Theory of Acceptance and Use of Technology.

Higher educational attainment was linked to a greater likelihood of being influenced by social factors to adopt the platform (AOR 1.847; 95% CI 1.232–2.770). Participants with higher education levels were also more likely to be motivated by effort expectancy to adopt the website (AOR 1.479; 95% CI 1.064–2.054), the smartphone app (AOR 1.412; 95% CI 1.045–1.909), and the CRM-based messaging system (AOR 1.375; 95% CI 1.010–1.872). Additionally, they were more likely to be motivated by perceived information security to use the smartphone app (AOR 1.625; 95% CI 1.143–2.310) and the CRM-based messaging system (AOR 1.510; 95% CI 1.045–2.183). They also tended to be influenced by facilitating conditions when using the smartphone app (AOR 1.438; 95% CI 1.073–1.927) and the CRM-based messaging system (AOR 1.530; 95% CI 1.110–2.110). Employed participants were notably more inclined to be encouraged by social influence to use the platform (AOR 11.167; 95% CI 1.354–92.110).

The earlier the year of first infection, the less likely participants were to be motivated by performance expectancy to use the platform (AOR 0.685; 95% CI 0.495–0.948). Participants who experienced more COVID-19 infections tended not to be driven by performance expectancy to adopt the platform (AOR 0.536; 95% CI 0.342–0.839), the smartphone app (AOR 0.652; 95% CI 0.427–0.995), and the CRM-based messaging system (AOR 0.644; 95% CI 0.419–0.990). They were also less likely to be encouraged by social influence to use the platform (AOR 0.486; 95% CI 0.295–0.802) and by facilitating conditions to adopt the website (AOR 0.637; 95% CI 0.418–0.971).

## Discussion

### Summary of findings

In this mixed-methods study, we aimed to understand the intentions to utilise our multi-component supported self-management platform to support rehabilitation among individuals with Long COVID-19. Through semi-structured interviews, we identified a total of 17 themes influencing the use of the platform and its components, categorised across the six Meta-UTAUT attributes (i.e., performance expectancy, effort expectancy, social influence, facilitating conditions, perceived information security, and perceived enjoyment). Particularly, nine of these themes exhibited a strong positive or negative influence. The cross-sectional survey indicated that each of the six Meta-UTAUT attributes influenced the intentions to use the platform and its components among over 70% of participants. Multivariable logistic regression analyses revealed that gender, age, educational attainment, employment status, the year of first COVID-19 infection, and the number of COVID-19 infections significantly predicted participants’ responses to specific Meta-UTAUT attributes.

### Strengths and limitations

This study employed a mixed-methods design, incorporating semi-structured interviews and a cross-sectional survey, to enable a comprehensive evaluation of users’ intentions to use the Long COVID-19 supported self-management platform and its various components, focusing on Meta-UTAUT attributes. However, it has several limitations. Firstly, this study did not aim to assess the validity or reliability of the Meta-UTAUT framework. Rather, we employed it as a theoretical framework to explore users’ intentions. However, should the platform be used by a sufficiently large sample, and if we are able to recruit a substantial sample in the future, it would be beneficial to conduct additional analyses, such as confirmatory factor analysis and calculation of Cronbach’s alpha coefficient,^20^ to further validate the framework. Second, we only included current users of the self-management platform in the interviews and cross-sectional survey. Without the participation of individuals who withdrew from or did not engage with the programme, not only would our findings be at risk of sampling bias, but we would also be unable to conduct subgroup analyses comparing current users and ex-users on their agreement with different Meta-UTAUT attributes. This limitation hinders our ability to identify which themes or attributes may contribute to early withdrawal. Third, we opted not to include all 10 Meta-UTAUT attributes to balance the questionnaire’s length against the marginal gain of additional information, and in response to feedback from the semi-structured interviews. Consequently, caution should be exercised when interpreting our findings, as they may not capture users’ intentions to utilise the self-management platform in a comprehensive manner. Fourth, while statistical associations were found between participants’ responses on Meta-UTAUT attributes and certain sociodemographic characteristics and COVID-19 infection history, providing relevant interventions for individuals with these characteristics may not necessarily alter their intentions to use the platform. Fifth, we did not include NGO case managers in the interviews, who might have assisted users in navigating the platform, nor healthcare providers or platform developers, who could have offered additional perspectives. This omission limits our ability to capture the full extent of external support that might have influenced users’ experiences and their responses to the survey. Furthermore, the cross-sectional design of this survey was unable to capture changes in users’ intentions over time. If resources permit, follow-up studies could be conducted to provide a longitudinal perspective. Finally, while our findings are specific to the Hong Kong context and may not be generalisable to other settings, this work provide a valuable example of how similar studies could be conducted in different contexts.

### Implications for practice

Supported self-management for Long COVID-19 is widely recommended by national and international authorities and institutions due to its effectiveness in improving the health conditions of affected individuals and its prospects for reducing healthcare expenditures^5–7^. Given the high prevalence of this chronic condition and the increasing demand for self-management support in Hong Kong, we developed an online Long COVID-19 supported self-management platform to provide the five categories of advice and information deemed essential by NICE as a self-management facilitator^6^. Specifically, our platform provided a range of self-management resources, including both textual and video content, encompassing approaches from both Chinese and Western medicine, as well as dietary and physical training recommendations, all of which are widely practised and accepted in the territory. The CRM-based messaging system on WhatsApp Messenger facilitated bidirectional communication between staff and users, allowing the exchange of textual and voice messages. This capability maximised support, enhanced user engagement, and provided a more interactive, responsive approach to help manage Long COVID-19 symptoms.

However, the implementation of this kind of supported self-management platforms must consider the barriers that certain populations, particularly older adults, face when using information and communications technologies for health support. Hearing and sight limitations, the need to remember passwords, and reduced fine motor control are prevalent barriers among older adults in using information and communications technologies for health support^21^. These barriers may lead to additional needs for this population and help explain our results from the regression analyses, which indicated that older adults tended to report not receiving enough assistance when using the website and the CRM-based messaging system. This lack of support may, in turn, contribute to their reduced enjoyment when receiving health information through these channels. Despite the dearth of facilitation conditions and perceived enjoyment, older adults constituted a relatively large group among all active users. As suggested by the interviews, future optimisation of our platform or other self-management programmes may prioritise person-centred interactions, in both written and verbal formats, on the CRM-based messaging system. Compassionate and empathetic staff may significantly enhance older users’ experiences, improving their satisfaction and engagement with the platform, which, in turn, makes the self-management process more enjoyable and intriguing for this population. Also, built-in user guides should be easily accessible—featuring simple and clear navigation, easy-to-understand text, appropriate font sizes, and audio options^22,23^—on similar websites for supported self-management, as recommended by the interviewees, to facilitate usage. If feasible, self-management facilitators may offer referral services through the online platform to connect older adults with appropriate online and in-person health and social care services, ensuring that they receive the support they need.

Moreover, it is essential to acknowledge that individuals with higher educational attainment generally possess higher digital health literacy^24–26^, alongside increased confidence and enhanced problem-solving skills within digital contexts^27^. These competencies might have contributed to their positive responses to the Meta-UTAUT attributes of effort expectancy, perceived information, and facilitating conditions. Considering their digital proficiency, authorities and institutions may consider investing in developing informative online platforms that offer comprehensive self-management recommendations for Long COVID-19 and other chronic conditions, supplemented by references to further in-depth resources. Empowering this population through such platforms could reduce their reliance on in-person care while improving overall health outcomes. Emphasising and demonstrating institutional credibility in promoting these platforms is also vital for fostering trust and confidence in the advice offered.

### Implications for research

In terms of research, future investigators evaluating supported self-management platforms for Long COVID-19 may recruit individuals with various common comorbidities, such as respiratory disorders, mental illness, and autoimmune conditions,^28,29^ to facilitate subgroup analyses regarding the intentions to utilise these services among diverse populations. Such analyses could inform the development of specialised promotion and support strategies aimed at maximising accessibility, service utilisation, user engagement, and overall user experience. Economic evaluations may also be conducted to assess the cost-effectiveness of supported self-management platforms compared to local multidisciplinary rehabilitation programmes from a health and social care system perspective. Such evaluations would enable analyses of incremental differences in public monetary costs relative to variations in health outcomes, such as quality-adjusted life years^30^. The resulting evidence would inform local policymakers on the most efficient allocation of public resources for managing Long COVID-19, ultimately supporting the development of locally adapted, evidence-based guidelines and ensuring that interventions are both effective and sustainable within the health and social care system.

## Conclusions

Supported self-management is an emerging intervention for Long COVID-19. While online platforms enhance access to health information, bi-directional communication through a CRM-based messaging system may maximise both professional and emotional support, thereby enhancing user engagement. Funding and efforts may be directed towards removing digital barriers for the older population and facilitating case referrals to relevant online and in-person health and social care services, ensuring they receive comprehensive support tailored to their needs.

## Electronic supplementary material

Below is the link to the electronic supplementary material.

Supplementary Material 1

## Data Availability

All data supporting the findings of this study are available within the paper and its Supplementary File.

## Abbreviations

AOR: Adjusted odds ratios
CI: Confidence intervals
CRM: Customer relationship management
ICST: Institute of Clinical Science and Technology
IQR: Interquartile ranges
Meta-UTAUT: Meta-analysis-based modified unified theory of acceptance and use of technology
NICE: National Institute for Health and Care Excellence
