# Supplementary material for: Understanding intentions to use a multi-component supported self-management platform for long COVID-19: a mixed-methods evaluation in Hong Kong
Source: Sci Rep. 2025 May 8;15:16086. doi: 10.1038/s41598-025-01239-0 (PMC12062295; doi:10.1038/s41598-025-01239-0)
Supplement: Supplementary file 1 — Supplementary Material 1 [file 41598_2025_1239_MOESM1_ESM.docx]

**Understanding intentions to use a multi-component supported self-management platform for Long COVID-19: A mixed-methods evaluation in Hong Kong**

**Supplementary file**

**Table S1.** Definitions of the Meta-UTAUT attributes

**Table S1.** Definitions of the Meta-UTAUT attributes

| **Meta-UTAUT attributes** | **Definitions** |
| --- | --- |
| **Performance expectancy** | The level to which an individual perceives that using the platform/component will help Long COVID-19 self-management. |
| **Effort expectancy** | The level of ease associated with the use of the platform/component. |
| **Social influence** | The level to which an individual’s important others believe that they should use the platform/component. |
| **Facilitating conditions** | The measure of infrastructural support available for use of the platform/component. |
| **Information security** | The level to which an individual perceives that their personal information is secure when using the platform/component. |
| **Perceived enjoyment** | The level to which an individual perceives that using the platform/component is fun, enjoyable, or intrinsically rewarding. |

Meta-UTAUT: Meta-analysis-based modified Unified Theory of Acceptance and Use of Technology
